# Sudden Unexpected Death in Alcohol Misuse—An Unrecognized Public Health Issue?

**DOI:** 10.3390/ijerph6123070

**Published:** 2009-12-04

**Authors:** Alexa H. Templeton, Karen L.T. Carter, Nick Sheron, Patrick J. Gallagher, Clare Verrill

**Affiliations:** 1 Liver Research Group, University of Southampton, Southampton General Hospital, Southampton University Hospitals NHS Trust, Tremona Road, Southampton, SO16 6YD, UK; E-Mails: karencarter@doctors.org.uk (K.L.T.C.); Nick.sheron@soton.ac.uk (N.S.); 2 Clinical Hepatology, Division of Infection, Inflammation and Immunity, University of Southampton Medical School, Southampton, SO16 6YD, UK; 3 Department of Histopathology, Southampton University Hospitals NHS Trust, Southampton, UK; E-Mails: P.J.Gallagher@soton.ac.uk (P.J.G.); clareverrill@hotmail.com (C.V.)

**Keywords:** alcohol, death, *post mortem* and sudden cardiac death

## Abstract

Sudden arrhythmic cardiac death can occur in chronic misusers of alcohol. The only findings at *post mortem* are fatty liver and a negative or low blood alcohol. This is an under-recognized entity. Coroner’s *post mortems* in a typical UK city were studied. Seven out of 1,292 (0.5%) *post mortems* were deemed to have died of alcohol associated arrhythmic death. Applying this study to the UK as a whole, alcohol related arrhythmic death or as we have termed it SUDAM (Sudden Unexpected Death in Alcohol Misuse) probably accounts for around 1,000 deaths, many of which are misattributed to other causes.

## 1. Introduction

Alcohol is estimated to be associated with approximately 40,000 deaths per year in the UK and deaths from alcohol related causes are increasing at an alarming rate, having increased at least 8-fold since the 1970s [1]. Due to recent media coverage, it is now accepted that increasing alcohol consumption is leading to huge increases in the numbers of deaths due to alcoholic liver disease. It tends to be forgotten however, that it is not alcoholic liver disease, but rather cardiovascular disease that is the most important cause of mortality in alcoholics [2]. What is not widely known is that those who chronically drink excess alcohol are, like epileptics, at increased risk of sudden death compared to the general population [3]. While this scenario is well described in epilepsy and termed Sudden and Unexpected Death in Epilepsy (SUDEP), this is not the case with alcohol, and we suspect that many deaths may be missed as a result.

It was first described in 1926 that there is an association between fatty changes within the liver due to alcohol and sudden (presumed) arrhythmic death [4,5]. These deaths typically occur in white males who are greater than 50 years old with a negative or low blood alcohol and the liver usually depicts fatty change rather than cirrhosis [6]. The mechanism of death is not fully understood, but thought to be due to a variety of metabolic disturbances triggered by massive ethanol intake and starvation [7] resulting in cardiac arrhythmia. *Post mortems* on these cases are essentially negative, showing only liver steatosis.

Alcohol causes arrhythmias during life, including prolongation of the QT interval, which is associated with sudden cardiac death [2]. One would logically infer from this that alcohol induced arrhythmias are a commonly stated cause of death. This, however, is not the case [3,8,9]. The fact that alcohol can be a cause of sudden cardiac death is unrecognized by many pathologists [8] and many of these deaths are being incorrectly attributed to other causes such as minor coronary artery atherosclerosis [10]. Studies of 10,353 middle aged males involved in a health screening program in Malmo, Sweden, found that alcohol related mortality was the commonest cause of death [11–13]. Of the 347 deaths in this program investigated in 1988 [10], 46 (0.4%) were sudden unwitnessed deaths with an alcohol risk and no pathological findings. In a UK survey of sudden deaths, 4.1% of deaths had no clear cause of death found at *post mortem* and a further 1.2% had no clear cause of death and a history of alcohol abuse [3]. This suggests that a proportion of sudden unexpected deaths are due to alcohol related arrhythmia, but are still not being recognized as such. These deaths are obviously upsetting for the family, resulting in unanswered questions and are also frustrating for the pathologist.

The presence of steatosis at *post mortem* is a sensitive test of high consumption of alcohol; moderate or severe fatty liver representing those who drink more than 80 g or 10 units of alcohol per day [14]. Although previous series of the scenario of sudden death in association with fatty liver in alcoholics have been published, these are mainly from outside of the UK and are published in languages other than English [9,15,16]. Many are also over 20 years old. With increasing alcohol consumption in the UK, we have absolutely no idea how many cases of sudden arrhythmic death are occurring in those who chronically drink alcohol to excess.

This study therefore sets out to identify the proportion of deaths at *post mortem* which are being attributed to alcohol related arrhythmia (*i.e*., with only fatty liver found at *post mortem*) and how many further deaths are being misattributed to other causes.

## 2. Methods

Ethical approval was obtained from the local ethics committee (Isle of Wight, Portsmouth and South East Hampshire Local Ethics Committee ref no: 07/H0501/71).

This study involved a prospective and retrospective study of adult post mortems at Southampton General Hospital during 2007 and 2008.

### 

#### Prospective Aspect:

Adult *post mortems* taking place at Southampton General Hospital between 8^th^ October 2007 and 14^th^ March 2008 were observed. Routine information on pathological findings in the heart, lungs and liver were documented, along with the cause of death. Details of past medical history including information on smoking, diabetes, hypertension and alcohol intake were recorded from information provided by the Coroner or where available from the General Practitioner or medical notes. Toxicology and liver histology results were also recorded if available. Data (unlinked, anonymised) was entered onto an SPSS database.

#### Retrospective Aspect:

All adult *post mortems* performed at Southampton General Hospital between 1^st^ January 2006 and 31^st^ January 2007 were assessed from computerized *post mortem* reports. The information recorded was as per the prospective aspect of this study. Any retrospective cases that were incomplete, outstanding or limited to the brain were excluded.

All cases with evidence of excess alcohol consumption during life were identified and separated from cases in which there was no evidence of this. The criteria used to identify this ‘*alcohol excess*’ group was adapted from that used previously by Petersson [10]. Inclusion into the alcohol excess group was by any one of the following:

1. a known history of excess alcohol consumption during life; either drinking >14 units per week for females, >21 units per week for males, or (as this kind of detailed information was seldom available), descriptions of being an “alcoholic” or “heavy drinker” provided by the Coroner’s office.
2. alcohol being directly involved in the chain of events leading to death, e.g., drowning in an alcohol-intoxicated state.
3. *post mortem* findings regarded as typical of alcohol misuse being present such as fatty liver (in the absence of diabetes mellitus or weight extremes (16.5 < BMI < 30) or alcoholic cirrhosis.
4. cases identified on toxicological analysis to show alcohol or alcohol related findings, e.g., alcoholic ketoacidosis.

All cardiac deaths in both the ‘alcohol excess’ (n = 162) and ‘non alcohol excess groups’ (n = 1130) were categorised using the Davies criteria [17], which is a well recognised system for estimating the probability of cardiac pathology present at *post mortem* causing death. Within the ‘alcohol excess’ group, deaths categorized as Davies criteria 3, 4, 5 (*i.e.*, the less certain deaths) or deaths which could not be accurately classified were reviewed by a consultant pathologist. This was to assess the evidence for the stated causes of death with the aim of confirming alcohol related arrhythmic deaths in the cases that were already stated as such and to identify further deaths in which alcohol related arrhythmia was probably the cause of death (and death was certified as something else).

Although alcohol can cause cardiac hypertrophy [10], cases with significant cardiac hypertrophy were excluded from being assigned as an alcohol related arrhythmia. Cases were excluded if there was (1) >20 mm thickness of left ventricle wall or a description of left ventricular hypertrophy was given in the *post mortem* report, (2) the heart weight was >340 g for females or >360 g for males or if the heart weight was above this and the features were otherwise classical of an alcohol related arrhythmia, body weight was taken into account and an increase of <30% heart weight above that expected for body weight (a method used previously by Davies) was allowed [18].The level of significance for coronary artery atheroma was taken to be the generally accepted level of a residual lumen of <1 mm (or >75% stenosed).

## 3. Results

### Demographics

A total of 1,312 cases were documented. 20 retrospective cases were excluded (19 as the *post mortem* reports were incomplete and one as the report was limited to the brain), giving a total of 1,292 cases that were examined (939 retrospective, 353 prospective). 782 (60.5%) were male, 509 (39.4%) were female and one retrospective case was of unknown sex. The median age of death was 74 years. 99.5% of *post mortems* were performed at the request of the Coroner. One hundred and twenty seven (9.8%) had a history of alcohol excess, 160 (12.4%) had diabetes mellitus and 305 (23.6%) had hypertension.

### Alcohol Excess Group

A total of 162 cases qualified for the ‘alcohol excess’ group, leaving 1,130 cases in which there was no suggestion of alcohol excess. These 162 cases included 127 cases with a history of alcohol excess during life. Within this alcohol excess group, males accounted for 113 cases (69.8%) and females for 49 (30.2%). By means of a Man Whitney U test the alcohol excess group was shown on average to have an age of death 12.1 years less than cases that showed no evidence of alcohol excess (p < 0.001, confidence intervals = 9.4–14.8), having a median age of death of 61 years. The causes of death in the ‘alcohol excess’ group are shown in Table 1. Forty of the 162 cases had liver histology taken as part of the routine *post mortem*. One case of alcoholic hepatitis was identified and given as the cause of death and this was based on clinical grounds (without histology being taken). This case was included in the category of ‘alcoholic liver disease’ deaths. Three of the 11 alcoholic liver disease deaths had liver histology taken and no cases of histological steatohepatitis were identified. The commonest single cause of death in the alcohol excess group was ischaemic heart disease (n = 27 or 16.7%). In the alcohol excess group, ‘coronary heart disease’ (*i.e.*, Davies criteria 1, 2 and 3 deaths) accounted for 32 or 19.8% of deaths *versus* 408 or 36.1% of deaths in the group with no history of alcohol excess.

All Davies criteria 3, 4 and 5 deaths in the alcohol excess group were reviewed. Four cases were identified in which death was certified as due to presumed (suspected) ventricular arrhythmia due to steatosis of the liver (or cirrhosis) plus or minus alcohol abuse and on review seemed to conform to the classic scenario previously described for alcohol related arrhythmic death. In addition, a further three cases were identified in which in the authors’ opinion, alcohol related arrhythmia was the probable cause of death rather than the stated cause of death. These seven cases showed the following features: all male, blood alcohol raised (but not to fatal levels in the two cases in which it was available) and nothing to find at *post mortem* except liver steatosis or cirrhosis (except one case showing congestion). These seven cases accounted for 0.5% of all deaths surveyed (or 4% of the deaths in the alcohol excess group) and the features are shown in Tables 2 and 3. A further 10 potential cases of alcohol related arrhythmia were identified, but these were excluded on the grounds of cardiac hypertrophy or other significant medical conditions that could have accounted for death.

### Sudden Cardiac Death

Table 4 shows the numbers of deaths in each of the Davies’ criteria groups 1–5 in the alcohol excess group *versus* the non alcohol excess group. In the alcohol excess group, there were relatively fewer deaths compared to the non alcohol excess cases in groups 1, 2 and 3, *i.e.*, the deaths where we can be more certain about the cause of death. Interestingly, there is an increased proportion of Davies criteria 5 deaths (4.3% of cardiac deaths versus 1.3% in the non-alcohol excess group). This is the group of deaths in which alcohol related arrhythmias will be present. In this study, five of our seven highlighted arrhythmic deaths were in Davies category 5. The other two cases were in category 4 and unclassified.

## 4. Discussion

Seven cases of presumed fatal arrhythmia in individuals who chronically drank alcohol to excess were identified The pathological findings were similar to those found in previous studies; namely that the deaths typically occurred in middle aged males, found dead at home and showed only liver steatosis [6]. In the two cases in which there was toxicology available, alcohol was present at non-fatal or low levels, as shown in previous studies [6,8]. These seven cases represented 0.5% of deaths undergoing coroner’s *post mortem*. In 2005, 230,000 deaths were referred to Coroners in England and Wales, accounting for 45% of all deaths [19]. Based on this study, alcohol related arrhythmia potentially accounts for 1,150 deaths in England and Wales each year.

A number of previous studies have examined the relationship between sudden death and fatty liver. Kuller *et al.* [20] surveyed all sudden cardiovascular and other non-traumatic deaths undergoing *post mortem* (n = 397) in one year in Baltimore, USA and found 28% were associated with fatty liver. May *et al.* [21] reported a rate of 9% in Pennsylvania, and Petersson 25% in Sweden [10]. In a further study from North Carolina, USA from 1972 to 1976 *post-mortems* were performed in 8% of 23,117 deaths, and 411 cases of fatty liver death were found [8,22]. The only study of fatty liver deaths conducted in the UK, was by Clark in 1988 [23] in Glasgow and it reported that 6% of 500 deaths in chronic alcoholics were associated with fatty liver.

The proposed mechanism of death in these cases remains elusive. Several explanations have been offered in the literature over the years. Although popular in the 1950s, fat embolism is now probably unlikely to be the cause in the majority of cases [8]. Alcohol withdrawal or hypoglycaemia were also thought to be possible mechanisms. Many alcoholics have a prolonged QT interval, which is associated with an increased risk of sudden death [2]. One study in which 11 patients actually made it for medical attention before dying of fatty liver sudden death found profound electrolyte disturbances including hypomagnesaemia and concluded that various metabolic disturbances triggered by the combination of massive ethanol intake and starvation was the most probable cause of these deaths [7]. Clark reported that in many of these deaths, that there was a history of feeling unwell the day or days before including vomiting, abdominal pain and fits [23]. Although not within the scope of this paper, it is supportive of a mechanism of death due to profound electrolyte disturbances resulting in fatal arrhythmia. In these older studies, there was no attempt to exclude deaths with cardiac hypertrophy or separate alcoholic ketoacidosis deaths from arrhythmic deaths. Although alcoholic ketoacidosis was originally described almost 70 years ago [24], it has only gained widespread recognition in more recent years. In this current study, alcoholic ketoacidosis (diagnosed by toxicology detection of blood • -hydroxybutyrate) was the single commonest alcohol related cause of death in the alcohol excess group (11%). In the authors’ experience, alcoholic ketoacidosis deaths can mimic sudden cardiac death, until the • -hydroxybutyrate reveals otherwise.

Toxicology was only available in this study on a limited number of cases, as only the extra studies requested as part of the routine post mortem were available. This is obviously a limitation of this study and it should be performed on all alcohol related deaths. In terms of establishing the presence of chronic alcohol misuse at *post mortem*, blood alcohol is generally not a useful marker as it falls to normal relatively rapidly after cessation of drinking and therefore only indicates the level of acute alcohol consumption. Possibilities for further research on these deaths in order to establish more reliable information on chronic alcohol consumption include interviewing relatives or friends of the deceased which is said to provide reliable information on drinking behaviour, but this is a difficult thing to do logistically, ethically and financially [14]. It can be done, however, and forms the basis of the psychological autopsy [25]. Another option is to screen *post mortems* for evidence of chronic excess alcohol consumption. This can be achieved by testing newly described biomarkers in blood or urine such as ethyl glucuronide, which can detect ethanol intake up to 80 hours after the blood ethanol level has fallen to zero [26].

Epileptics, like alcoholics, have an increased risk of sudden death compared to the general population [3] and recognition of this syndrome has increased since the acronym SUDEP (Sudden and Unexpected Death in Epilepsy) was coined. For example SUDEP accounted for 0.8% of deaths in this study. We believe that the use of a similar term SUDAM (Sudden Unexpected Death in Alcohol Misuse) would increase recognition of the syndrome amongst pathologists and lead to more accurate death certification, thus enabling the public health implications to be determined and trends to be analysed. A suggested definition of SUDAM would be: *‘sudden, unexpected, unwitnessed or witnessed, non-traumatic deaths in patients with a history of chronic excess alcohol consumption and or evidence of hepatic steatosis or other alcoholic liver disease where post mortem examination does not reveal a toxicological (specifically alcohol intoxication or alcoholic ketoacidosis are excluded) or anatomical cause of death and there is no significant cardiac hypertrophy’*. It is recommended that as a minimum these cases have a full *post mortem* including histology of the myocardium and liver with toxicology samples taken for blood alcohol and blood ketones (in particular • -hydroxybutyrate). Essentially like SUDEP, this should be a diagnosis of exclusion.

The relationship between alcohol and coronary heart disease has been the subject of recent media coverage in the UK due to a study published by Arriola *et al.* which found that moderate, high or very high alcohol intake by men reduces their risk of coronary heart disease by more than 30% [27]. Interestingly, despite this, ischaemic heart disease in this study was still the single commonest cause of death in the alcohol excess group accounting for 16.7% of deaths. Although not within the scope of this paper, it is also interesting that deaths due to coronary heart disease in the alcohol excess group were only half of that seen in the group with no history of alcohol excess (19.8% versus 36.1%), which would seem to support the Arriola *et al.* study. In addition to the seven cases labeled as SUDAM in this study, a further 10 cases were identified which were strongly considered as potential SUDAM cases, but were excluded on the basis of other pathologies being present, which could have accounted for death and this was usually cardiac hypertrophy or ischaemic heart disease. It may be that some of these 10 cases were indeed SUDAM cases, but at the present time, the criteria for SUDAM need to remain strictly defined until such time as it is more fully understood, allowing the boundaries to be widened. It is also likely in the authors’ opinion that in patients with preexisting cardiac disease (hypertrophic or ischaemic) that alcohol acts synergistically to potentiate fatal arrhythmia in some cases. There is just simply not enough data on this at this time from this study to answer this conclusively.

There are inevitable limitations of a *post-mortem* study performed under current patterns of practice in the UK. Nevertheless, we believe that our study has demonstrated that fatal arrhythmia in association with fatty liver and chronic excess alcohol consumption is a significant public health issue for the UK. It may account for around 1,000 deaths *per annum* in England and Wales with many of these deaths currently misattributed to other causes or simply unexplained. With the current trend for escalating alcohol abuse, in particular binge drinking, in the UK, much of the previous literature on this topic (which is decades old and not from the UK) is now not relevant. These deaths need accurate certification so that the trends in true prevalence can be monitored.

## Figures and Tables

**Table 1.** A breakdown of causes of death for the “alcohol excess” group (n = 162) divided into cardiac and non cardiac causes.

**Cause of Death (Cardiac)**	**Number of cases**	**Cause of Death (Non-Cardiac)**	**Number of cases**
Ischaemic heart disease	27	Alcoholic ketoacidosis	17
Cardiac failure	5	Bronchopneumonia	13
Ventricular arrhythmia due to fatty liver	4	Gastrointestinal bleed (non-variceal)	13
Dilated cardiomyopathy	4	Trauma*	11
Hypertensive heart disease	4	Alcoholic liver disease**	11
Ischaemic and hypertensive heart disease	3	Drug overdose / toxicity	8
Myocardial infarction (including coronary artery thrombosis)	3	Intracerebral haemorrhage or infarct	7
Hypertensive heart disease and alcohol toxicity	1	Alcohol intoxication +/− drug toxicity	5
Hypertensive heart disease and alcoholic liver disease	1	Pulmonary embolism	2
Pericarditis	1	Epilpsey/SUDEP	2
Aortic stenosis	1	Other***	19
**Total**	**54**	**Total**	**108**

* including carbon monoxide poisoning, burns, hanging, drowning.

** including cirrhosis, alcoholic hepatitis, variceal bleeding.

*** sickle cell crisis, small bowel ischaemia, metastatic carcinoma, perforated diverticulitis, pyelonephritis, ruptured abdominal aortic aneurysm, amyloidosis, mesothelioma.

**Table 2.** Causes of death and post mortem findings in the seven cases of alcohol related arrhythmia.

**Case**	**Age**	**Sex**	**Location of death**	Heart weight (g)*	Appearance of liver **	Blood alcohol mg/100 mL***	**Stated cause of death**
1	60	M	Community	350	Steatosis		1a Presumed ventricular arrhythmia 1b Fatty change of the liver
2	84	M	Community	380	Steatosis (grade 3)	351	1a Pulmonary oedema 1b Presumed ventricular arrhythmia 1c Fatty change of liver, alcohol abuse
3	53	M	Community	350	Cirrhosis, steatosis	176	1a Acute cardiac failure 1b Coronary artery atherosclerosis
4	51	M	<24hrs in hospital	280	Congestion		1a Acute pulmonary oedema 1b Cardiac failure
5	59	M	Community	442	Congestion, steatosis.		1a Presumed cardiac arrhythmia 1b Cardiac failure
6	46	M	Community	405	Cirrhosis		1a Suspected ventricular arrhythmia 1b Liver failure 1c Cirrhosis of the liver
7	39	M	Community	352	Steatosis		1a Suspected ventricular arrhythmia 1b Severe fatty change of liver

*Key to Table*:

* An approximately normal heart weight would be 280–340 g (females), 320–360 g (males).

** Liver appearance based on macroscopic appearance unless histology available in which case this is recorded (cases 2, 3, 5 had liver histology available).

*** If available as part of the routine *post mortem*.

**Table 3.** A table showing a summary of the seven cases selected on review to show features suggestive of an alcohol induced arrhythmia. Basic demographic features of the cases are shown in Table 2.

**Case**	**Rationale for inclusion**
1	There was a history of excess alcohol consumption. This case shows the ‘classic’ features of an alcohol associated arrhythmia.
2	There was a history of excess alcohol consumption. This case shows the ‘classic’ features of an alcohol associated arrhythmia. Blood alcohol was high, but probably not sufficient to account for death in someone with tolerance.
3	The deceased was known to consume excess alcohol. Grade 3 steatosis and cirrhosis were present. Death was attributed to coronary artery atherosclerosis, but there were no coronary arteries with a luminal diameter of <1mm and there was no myocardial fibrosis. Death could have been due to alcohol induced arrhythmia.
4	The deceased was known to consume excess alcohol and suffered from diabetes mellitus. Severe pulmonary oedema was present suggesting an arrhythmic death. No obvious cardiac abnormalities were present. Death could have been due to alcohol associated arrhythmia.
5	No information on alcohol consumption was available, but hepatic steatosis was present on histology. Moderate pulmonary oedema suggested an arrhythmic death. The heart was 442 g and the body weight was 74 kg. The predicted heart weight for this body weight would be approximately 400 g and allowing for up to 30% increase in heart weight we can be fairly confident that there was no significant cardiac hypertrophy. No left ventricular hypertrophy was described at post mortem. Death could have been due to alcohol associated arrhythmia.
6	The deceased was known to consume excess alcohol. Severe pulmonary oedema was present, suggestive of an arrhythmic death. This case shows the ‘classic’ features of an alcohol associated arrhythmia.
7	There was a history of excess alcohol consumption. Moderate pulmonary oedema was present. This case shows the ‘classic’ features of an alcohol associated arrhythmia.

**Table 4.** A table showing a description of the specific categorisation of sudden cardiac deaths (Davies’ Criteria) and the number/percentage of cardiac deaths in the group demonstrating evidence of excess alcohol consumption versus those with no evidence of this.

**Davies’ Criteria**	**Description**	**Number/percentage of cases where there is no evidence of alcohol excess**	**Number/percentage of cases within alcohol excess group**
**1**	Coronary atheroma and clear evidence of coronary thrombosis and/or acute myocardial infarction—*very high probability of causing sudden death*	146 (12.9%)	9 (5.6%)
**2**	Coronary atheroma with at least one coronary artery <1mm diameter and evidence of healed myocardial infarction—*moderate to high probability of causing sudden death*	155 (13.7%)	11 (6.8%)
**3**	Coronary atheroma with at least one coronary artery <1mm diameter, but no evidence of healed myocardial infarction—*questionable probability of causing sudden death: depends on number of stenosis and circumstances of death*	107 (9.5%)	12 (7.4%)
**4**	No evidence of ischaemic heart disease, but evidence of congestive heart failure or significant left or right ventricular hypertrophy and/or dilatation—*moderate probability of causing sudden death; cardiomyopathies should be excluded*	120 (10.6%)	14 (8.6%)
**5**	No significant cardiac pathology / unexplained sudden cardiac death (SADS) —*histology and toxicology essential if SADS is to be considered seriously*	15 (1.3%)	7 (4.3%)
**Cardiac Death (Not Classified)**	Cardiac death where insufficient information was available to be catagorised into Davies’ criteria 1–5	17 (1.5%)	3 (1.9%)
**Non Cardiac Death**		570 (50.4%)	106 (65.4%)
**Total cases**		1130	162
